# A qualitative study on caretakers' perceived need of bed-nets after reduced malaria transmission in Zanzibar, Tanzania

**DOI:** 10.1186/1471-2458-12-606

**Published:** 2012-08-03

**Authors:** Netta Beer, Abdullah S Ali, Helena Eskilsson, Andreas Jansson, Faiza M Abdul-Kadir, Guida Rotllant-Estelrich, Ali K Abass, Fred Wabwire-Mangen, Anders Björkman, Karin Källander

**Affiliations:** 1 Division of Global Health (IHCAR), Department of Public Health Sciences, Karolinska Institutet, Stockholm, Sweden; 2 Malaria Research Unit, Department of Medicine Solna, Karolinska Institutet, Stockholm, Sweden; 3 Zanzibar Malaria Control Programme (ZMCP), Ministry of Health, Zanzibar, Tanzania; 4 Department of Psychology, Stockholm University, Stockholm, Sweden; 5 Health Promotion Unit, Ministry of Health, Zanzibar, Tanzania; 6 EAP Canet Unitat Docent, Girona, Spain; 7 Healthcare Research Support Unit Metropolitana Nord. IDIAP, Barcelona, Spain; 8 Makerere University School of Public Health, Kampala, Uganda; 9 Malaria Consortium Africa, Kampala, Uganda

**Keywords:** Sub-Saharan Africa, Zanzibar, Malaria, Bed-nets, LLINs, Health belief model, Qualitative

## Abstract

**Background:**

The elimination of malaria in Zanzibar is highly dependent on sustained effective coverage of bed-nets to avoid malaria resurgence. The Health Belief Model (HBM) framework was used to explore the perceptions of malaria and bed-net use after a noticeable reduction in malaria incidence.

**Methods:**

Nineteen in-depth interviews were conducted with female and male caretakers of children under five in North A district, Zanzibar. Deductive content analysis was used to identify meaning units that were condensed, coded and assigned to pre-determined elements of the HBM.

**Results:**

Awareness of malaria among caretakers was high but the illness was now seen as easily curable and uncommon. In addition to the perceived advantage of providing protection against malaria, bed-nets were also thought to be useful for avoiding mosquito nuisance, especially during the rainy season when the malaria and mosquito burden is high. The discomfort of sleeping under a net during the hot season was the main barrier that interrupted consistent bed-net usage. The main cue to using a bed-net was high mosquito density, and children were prioritized when it came to bed-net usage. Caretakers had high perceived self-efficacy and did not find it difficult to use bed-nets. Indoor Residual Spraying (IRS), which was recognized as an additional means of mosquito prevention, was not identified as an alternative for bed-nets. A barrier to net ownership was the increasingly high cost of bed-nets.

**Conclusions:**

Despite the reduction in malaria incidence and the resulting low malaria risk perceptions among caretakers, the benefit of bed-nets as the most proficient protection against mosquito bites upholds their use. This, in combination with the perceived high self-efficacy of caretakers, supports bed-net usage, while seasonality interrupts consistent use. High effective coverage of bed-nets could be further improved by reinforcing the benefits of bed-nets, addressing the seasonal heat barrier by using nets with larger mesh sizes and ensuring high bed-net ownership rates through sustainable and affordable delivery mechanisms.

## Background

In 2009, malaria killed approximately 781,000 people [1], mainly children under the age of five living in sub-Saharan Africa (SSA), a region where malaria is responsible for an estimated 16 % of child deaths [2]. In the past, Zanzibar has been malaria endemic with high transmission of *Plasmodium falciparum* peaking during, or right after, the long rains. However, surveillance data show that malaria incidence has decreased dramatically in recent years after implementation and up-scaling of effective malaria control interventions [3-5].

In malaria-endemic countries, prevention of malaria largely relies on the use of insecticide-treated nets (ITNs), which have been shown to reduce overall child mortality by 18 % [6]. In Zanzibar, the decrease in malaria incidence was attributed to implementation and reinforcement of ITN interventions, as well as the provision of free artemisinin-based combination therapy (ACT) in public health facilities, and indoor residual spraying (IRS) in residential homes [3]. In 2005–2006 the Zanzibar Malaria Control Programme (ZMCP) carried out a mass campaign where long-lasting insecticidal nets (LLINs) were distributed free to all pregnant women and children under five. The distributed nets were blue rectangular Olyset® nets, made of polyethylene and with a large mesh size of 4 × 4 mm, and were impregnated with insecticide that lasts for at least five years. A survey that was carried out in the North A district approximately four months after the distribution revealed that the LLIN usage among children under five was high (87 %) and equitable [7].

As a result of the dramatic reduction in malaria, ZMCP is now advancing one step forward from the current "sustained control" effort to the “malaria elimination” target. One major challenge in achieving malaria elimination in Zanzibar is to prevent malaria resurgence [4]. Therefore, in addition to an efficient case detection and surveillance systems, it is crucial that vector control measures are maintained at a high effective coverage. It was shown that high effective coverage of LLINs largely relies on good access, as well as adherence to their use [7].

Previous studies have shown that the predicting factors that cause variation in ITN use are seasonality [8-11], age [10,12,13] and practical factors such as difficulties in mounting the nets and sleeping arrangements [10,14,15]. The belief that ITNs have only partial effectiveness, and the fear of insecticide toxicity, were also identified as factors that may hinder bed-net usage [16]. In some studies, the specific characteristics of the bed-nets such as condition, shape and type of the bed-net were also found to affect usage [14,17]. In Zanzibar, LLIN use was significantly higher among children whose caretakers perceived the LLINs to be better than conventional nets, and in children who received an LLIN that was assigned to them during the distribution [7]. Local perceptions and beliefs about a disease and its prevention can also determine adherence to malaria preventive and treatment interventions [18-20].

In an attempt to better understand health-related behavior and the determinants of adherence to health interventions, a number of theoretical models have been proposed. The Health Belief Model (HBM) is one of the most widely used social cognition models to study and promote the uptake of health services and predict health behavior. Although it was originally developed in the 1950s to explain low participation in medical screening programs, today it is used for a broad spectrum of health-related behaviors. It has been used in HIV/AIDS research, mainly for understanding risk behaviors [21], and has also been used in the field of malaria to understand use of malaria prophylaxis among travelers [22,23].

The HBM states that, in the case of prevention, individuals will take a health related action if they have a desire to avoid an illness and if they believe that a specific health action will prevent the illness. The model includes six elements: 1) **Perceived susceptibility** of the individual to the condition; 2) **perceived severity** of the condition as having serious medical and social consequences; 3) **perceived benefits** of taking the health action in reducing the disease threat as well as other additional benefits; 4) **perceived barriers** to taking the health action, which should not overweigh the benefits. These four perceptions are elements that determine the readiness to take the action. They are activated by: 5) **Cues to action** which trigger this readiness and 6) **self efficacy,** which is the conviction that one can successfully execute the health behavior [21,24].

Given that the aim of eliminating malaria in Zanzibar is highly dependent on sustained effective coverage of bed-nets, it is important to understand the community risk perceptions about the disease and how the perceived need for continued malaria prevention behavior is influenced by the significant reduction in malaria observed in Zanzibar in recent years. In this study, the HBM framework was used to explore the perceptions and beliefs of caretakers of children under five years who had received a free LLIN 13–14 months prior to the study.

## Methods

To explore caretakers' perceptions and beliefs about malaria and bed-nets, a qualitative approach was deemed to be most appropriate. The Health Belief Model (HBM) was used to guide the data collection and analysis in order to explore the different perceptions about malaria and bed-net use.

### Study setting

Zanzibar consists of two large islands, Unguja and Pemba, and numerous small islands. The climate is tropical and humid, with two distinct rainy seasons: the long or heavy rains (Masika) between March/April and May/June and the short rains (Vuli) between October and December. There is also a seasonal classification by temperature, whereby the cool season occurs from June to November and the hot season from December to March.

The estimated total population of Zanzibar in 2007 was over 1.1 million with approximately 65 % on Unguja Island and 35 % on Pemba. Under-five children comprise around 16 % of the total population. The population is mostly Muslim and the spoken language is Kiswahili. The majority of the population is rural and the main livelihood is subsistence farming and fishing. The study was conducted on Unguja island, in North A district.

As mentioned in the background section, malaria transmission has rapidly declined in Zanzibar. From being a high-transmission area, it is now considered a low-transmission area and is moving towards malaria elimination.

### Sampling and study participants

The study team consisted of Swedish and Zanzibari specialists in the fields of public health, psychology and malaria. The interpreters who were used were fluent in both Kiswahili and English and had prior experience in translation work.

The interviews were carried out in February and March 2007. The informants were chosen purposefully to fulfill the following criteria: (1) residents of the North A district; (2) primary caretakers of children who were under the age of five at the time of the LLIN distribution and (3) recipients of at least one free LLIN in the preceding mass distribution campaign 13–14 months earlier. The informants were selected using data from a previous household survey about bed-net use in the North A district which was carried out about nine months earlier. During that household survey, 264 households from North A district were selected using two-stage random sampling, and one caretaker was interviewed in their home by a trained interviewer using a structured questionnaire with closed and open-ended questions [7]. Users and non-users of LLINs according to the household survey, as well as female and male caretakers were chosen to ensure maximum disparity. Respondents who had given extensive answers in the open-ended questions in the survey were prioritized to increase the likelihood of selecting interviewees who were more open and talkative. In a small number of cases, the requested informant was not available for interview and a different informant from the same village who met the inclusion criteria was selected instead.

Twenty interviews were scheduled to start with. Following each interview, the translated transcripts were reviewed to assess whether more interviews needed to be scheduled or whether saturation was achieved, as suggested by Dahlgren et al.…, 2007 [25]. After 19 interviews, it was felt that saturation had been reached.

First contact with the chosen informants was handled through the Shehas (village leaders), who were given the names of the prospective informant a few days before the interview. The Shehas located the prospective informants, asked for their consent and informed them about the timing of the interview. On the day of the interview, the Sheha guided the research team to the informant's house.

Although both users and non-users of LLINs were chosen, it became clear during the interviews that some usage patterns had changed since the household survey took place, and all but two informants stated using LLINs at the time of the interviews (Table 1). The change in usage patterns since the household survey was not investigated, but most likely has to do with the time elapsed since distribution.

**Table 1:** Bed-net usage patterns

**Caretaker number**	**Gender**	**Age**	**Bed-net usage by age**	**LLIN usage by age**	**Bed-net usage by season**
1	Female	44	Everyone	Mother and small child	Continuous
2	Female	35	Only children	Children	Seasonal
3	Female	35	Everyone	Mother and children	Unclear
4	Female	70	Everyone	The child	Unclear
5	Female	26	Everyone	The baby	Seasonal (continuous use only for baby)
6	Female	35	Everyone	Children	Continuous
7	Female	28	Everyone	Children	Seasonal
8	Female	40	Everyone	Children	Continuous
9	Female	33	Everyone	The child that got the net (away from home)	Continuous (for mother and child) and seasonal (for others)
10	Female	27	Everyone	Everyone	Continuous
11	Male	28	Everyone	Children	Seasonal
12	Male	50	Only children	Children	Seasonal
13	Male	36	Only the youngest child (3-year-old)	The youngest child	Seasonal
14	Male	53	Everyone	Children	Continuous
15	Male	52	Everyone	Young children	Partly seasonal (more continuous for children)
16	Male	55	Everyone	Not using the LLIN	Continuous
17	Male	27	Only the children	Not using the LLIN	Continuous
18	Male	47	Everyone	Children	Continuous
19	Male	45	Everyone	Mother and young children	Seasonal (for adults) and continuous (for children)

### Data collection

The interview guide was structured to capture the different elements of the Health Belief Model (HBM), and included questions on perceived susceptibility and severity of malaria, benefits and barriers of bed-net usage, cues to action and self-efficacy in using bed-nets. The interpreters were introduced to the interview guide, and translated it from English to Kiswahili. The interview guide was pilot tested on two informants and checked for construct validity by reviewing the answers given by the informants and assessing their relevance to the different HBM elements. Minor adjustments were made after the pilot testing and throughout the study based on experiences from previous interviews. Additional issues were also discussed, such as family background, intra-household relations, other preventive strategies and health-seeking behavior. The interview guide can be made available on request from the first author.

A female researcher and interpreter conducted the interviews with women, while a male researcher and interpreter interviewed the men. The questions were asked in English by the researchers and translated into Kiswahili by the interpreters. The informant's answers were then translated by the interpreters from Kiswahili to English for the researchers to understand and be able to formulate follow-up questions. The interviews lasted between 45 and 60 minutes, were conducted in or just outside the informants' houses and were recorded using digital voice recorders. The interviews were transcribed in Kiswahili by the interpreter, and the transcriptions were then translated into English and reviewed by the researchers, following each interview. Any unclear items were discussed with the interpreter and misunderstandings were clarified.

The term used for describing malaria was "homa ya malaria" (malaria fever). This term was well understood by the informants and interpreters, as was evident from the description of symptoms and link to mosquito bites. Other forms of fever ("homa") were also discussed and compared with the specific malaria fever; however in this article we present and focus on findings relevant to the malaria disease.

### Analysis

Content analysis was used to analyze the data. The HBM served as the main framework for this study and its elements served as the categories. Therefore, a deductive (directed) approach was applied whereby key categories were pre-determined according to the theory used (i.e. perceived susceptibility, perceived severity, perceived benefits, perceived barriers of bed-net usage, cues to action, and self efficacy) [26,27]. Each interview transcript was read several times, and meaning units were extracted independently by three of the researchers. Meaning units were copied into a matrix where they were condensed and assigned a code [28], which was then placed under a pre-determined HBM category. The three researchers then compared the codes under each category, and came to an agreement on the analysis outcome.

Ethical approval for the study was obtained from the Zanzibar Medical Research Task Force. In addition, a meeting was held before the start of the study where the Shehas for each village involved were informed about the purpose of the study. Written consent was obtained from the informants before starting the interviews.

## Results

Nineteen interviews were conducted. Out of the 10 female informants, nine were mothers aged 26–44 and one was an elderly 70-year-old aunt. The women's education level varied from no formal education to secondary education. All mothers worked in farming in addition to their household chores and most were also engaged in small-scale home-run businesses. The male informants were 27–55 year old fathers, and two of them were also caring for their nephews. Six were working as farmers, fishermen or daily laborers, while three were professionals (artist, retired soldier and an employee of the ministry of agriculture).

The findings are presented under the Health Belief Model (HBM) element clusters, i.e. ‘susceptibility and severity of malaria’, ‘benefits and barriers to bed-net usage’ and ‘self-efficacy and cues to action’.

### Susceptibility and severity of malaria

Caretakers generally felt that malaria was no longer a common disease. However, perceived susceptibility was strongly linked to mosquito density, and was considered higher when mosquitoes were more prevalent. Susceptibility was also considered greater in children.

"I pity the child because an adult can at least hit it [the mosquito] and it will leave, but a child is not able to do that….A child's health is delicate so he can get infected quickly" (Informant 17).

Prevention activities were suggested as the reason for the reduction in malaria burden felt by the caretakers interviewed. Thus, the perceived susceptibility to malaria while using vector control measures, especially bed-nets, was considered very low. Malaria medication and the provision of intermittent preventive treatment (IPT) to pregnant women were also mentioned as reasons for malaria reduction. While caretakers had experienced malaria in the past, it was often long since they or their children had had a malaria episode.

"We will not get malaria because we protect ourselves and it has been long since we had it … Like her [3-year-old child], she has not had that kind of fever [malaria] since she was born" (Informant 6).

Despite the risk reduction, caretakers thought that there was still a risk of contracting the disease if, for example, the mosquitoes bite through or from within the net, if malaria is contracted outside the house, or if God wishes.

"Sometimes it [mosquito] penetrates the net, or without penetrating you may lean or touch the net, then the mosquito bites you from outside" (Informant 12).

Caretakers viewed malaria as a mild illness that can easily be cured if timely and appropriate treatment is given, and were confident that they could easily access this treatment. However, they also acknowledged the fact that severe complications might develop if proper treatment is delayed and that children with malaria needed to be taken urgently to hospital for appropriate medication. Among its complications, malaria was known to develop into a mental problem, cerebral malaria, convulsions and fits, which could sometimes cause disability or death. The severe form of malaria was considered dangerous and even life-threatening, especially for young children.

"There is danger because if she [the child] continues to have fever and getting irritable she will have severe fever which might turn to another fever. The convulsions, stiffness, death will happen." (Informant 10).

Other consequences of malaria were the costs involved in seeking and obtaining care and the disruption of the family's daily life and routines. The caretakers explained that they would not be able to go on with their daily activities because they would have to seek care and look after the sick children. Economic consequences when a child was ill included spending money on treatment seeking, diagnosis and treatment as well as loss of work-days. Emotional consequences as a result of malaria were also mentioned, as caretakers would be sad and worry about the sick child.

"When my child was sick I was feeling very uncomfortable because I couldn't work; you are forced to concentrate on the child and saving the child from illness" (Informant 16).

### Benefits and barriers to bed-net usage

The caretakers acknowledged the benefit of bed-nets as protection against malaria, as well as other vector-borne illnesses. Caretakers also reported that using bed-nets had helped the family's well-being in general and that they and their children did not get ill as often when the bed-nets were being used.

The important benefit of bed-nets in the protection against mosquito nuisance and other insects was also highlighted. Caretakers mentioned that the mosquitoes disturbed sleep by biting and making noise.

"I think it is good [to use a bed-net] because the mosquitoes cannot penetrate. During the night I just enter into it and sleep well. If you do not cover yourself with a bed-net, you will not be able to sleep. You will have to chase the mosquitoes away here and there, they bite you sometimes, but if you sleep with it you will get a very nice sleep without problems." (Informant 1).

Caretakers believed that bed-nets were even more effective when treated with an insecticide. The LLINs were thought to have additional advantages including their wider square shape which covers the bed properly, the strong texture that does not tear easily and the large mesh-size allowing for more ventilation. However, big mesh-size was also mentioned as a disadvantage due to the fear that mosquitoes would penetrate the net, and the square shape was mentioned as difficult to mount. Caretakers stated that the insecticide of the ITNs and LLINs only affect malaria-carrying mosquitoes but not the other mosquito types.

**Figure 1:**
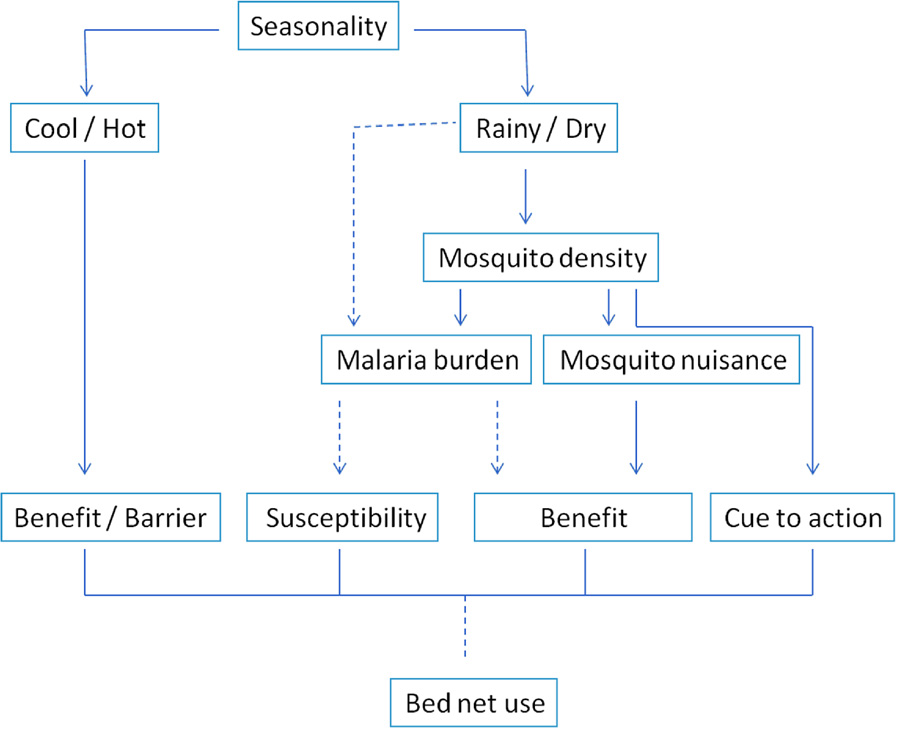
The effect of seasonality on elements of the health belief model.

One factor that clearly reduced consistent bed-net use was seasonality, whereby usage was stated to vary due to temperatures and rainfall. Figure 1 shows the different ways in which seasonality was perceived to have an effect on the different HBM elements. While the discomfort of sleeping under a net in the hot season was perceived as a barrier, in the cold season keeping warm under the net was seen as a benefit. Additionally, in the rainy season mosquito density was perceived to be higher, affecting both the perceived intensity of malaria burden and mosquito nuisance. It remained unclear, however, whether seasonality affected the perceived malaria burden directly or solely through mosquito density. The mosquito nuisance affects the perceived added benefit of the bed-nets as protection against mosquito bites. The malaria burden might have an effect on the perceived susceptibility to malaria and the perceived benefit of using the net as protection against malaria, although this was not clearly stated.

"[I use bed-nets] Everyday, during the rainy seasons, except for the very hot seasons …as is too hot, and there are no mosquitoes" (Informant 5).

Although all caretakers received a free LLIN during the distribution campaign, it was felt that these nets were insufficient and that they would eventually need to be replaced once they wore out. The cost of bed-nets was mentioned as a barrier to bed-net ownership as the cost of 5,000 Tanzanian shillings (3 US dollars) for a net was seen as unaffordable.

While caretakers’ awareness of the link between mosquitoes and malaria was high, female caretakers in particular had strong beliefs in alternative causes of malaria which potentially could reduce the perceived benefit of bed-nets as a malaria prevention method. Apart from mosquitoes, malaria was also perceived to be caused by dirty surroundings and dirty water as well as eating dirty things, having a bad diet and being hungry. Prevention for these causes included keeping surroundings clean and removing rubbish or water ditches, not allowing children to play with dirty water, providing the children with a good diet and caring for their personal hygiene. Religious beliefs about the role of God and spirits in relation to malaria and illness included the idea that mosquitoes are brought by God or that malaria is caused by bad spirits. Belief in spirits often coincided with the interpretation that malaria symptoms should be treated by a traditional healer.

"If someone gets malaria, it [care-seeking behavior] will depend on the way the people think. Some will tell you 'let us take him to the hospital. Why is he like this today? Why is he shouting? Why is he doing like this?' But others will say 'it is the devil so let us take him to the traditional healer'. Now everyone has his own way of thinking" (Informant 1).

All interviewed caretakers had had indoor residual spraying (IRS) done in their homes at least once. IRS was often perceived to be a useful way of killing mosquitoes and other insects such as cockroaches and bed bugs. However, the reduction in insects and mosquitoes was sometimes perceived as short-lived.

"[After IRS] for a few days it was better. Even those [mosquitoes] who are just flying around were few. But nowadays they are as many as before" (Informant 8).

Another drawback of IRS which was mentioned was the belief that IRS only kills malaria-carrying mosquitoes. Although the caretakers stated that they were not able to differentiate between malaria-carrying mosquitoes and other mosquitoes, they were told that if mosquitoes remain after spraying, they were not mosquitoes that can cause malaria.

"According to what the sprayers say, those normal mosquitoes survive the spraying but the malaria-carriers die" (Informant 18).

### Self-efficacy and cues to action

Clear separate roles between men and women were described, whereby women were mainly responsible for household chores, caring for children and farming activities, and men were responsible for providing livelihood and decisions on purchases. While male informants claimed to be the main decision-makers in all matters concerning the household and children, the women also claimed that they were quite independent with regard to issues concerning raising the children, as the fathers were often away from the home. Some responsibilities and decisions were shared, such as farming activities, some purchasing decisions (including bed-nets), bed-net usage and other issues concerning the children's health.

Female caretakers were mostly the ones responsible for covering the children with a bed-net at night, and self-efficacy of bed-net use was reported to be high, as this was an activity they mentioned they could easily master and control. Caretakers reported that they did not encounter any difficulties in using the bed-nets and that it had become part of every caretaker's routine to arrange the bed-nets for the children when putting them to bed.

"If the mosquitoes are there, it is easy to bring the net down. If there are no mosquitoes, then it is also not difficult to fold it up" (Informant 5).

Another indication of high self-efficacy in female caretakers was due to their more frequent exposure to health information through the health facilities.

"Mostly it is the women who get these instructions in the hospital at the time of pregnancy or even before or after delivery…. Once the mother has gotten instructions and advice about the children from hospital, we just sit down and discuss" (Informant 19).

Despite the high perceived self-efficacy, there was an indication of a strong dependency on the government to provide and re-treat bed-nets. Although caretakers stated that they could take these actions on their own, they reported that they often rely on the government to provide them with malaria control interventions.

The main cue to using bed-nets was the increase in mosquito burden, both seasonal (Figure 1) and the observed general increase in recent years.

"[We started using bed-nets] to protect us from mosquitoes. Before there were no mosquitoes in our village… you could sleep anywhere without seeing a mosquito, but now people have constructed big houses with many sewage tanks. That's why the mosquitoes have started to breed, there used to be no mosquitoes in our village" (Informant 9).

Other cues to bed-net use included being told to do so by health workers or after hearing about malaria in the media. The age of the child was also a cue to action whereby young children were prioritized when there were not enough bed-nets in the house. Parents, especially the mother, would often share a bed-net with a child. Children were also more likely to have continuous, rather than seasonal, bed-net use. The caretakers were especially aware of, and complied with, the fact that the LLINs given to them in the free distribution were intended for their under-five children.

"We say it is the small children's right [to use LLINs] because they were especially given to help them" (Informant 15).

## Discussion

This study indicates that the overarching perception among caretakers was that although malaria was seen as an easily curable and uncommon illness, bed-nets were still valued for avoiding mosquito nuisance, especially during the rainy season. Thus, despite the dramatic reduction in malaria burden in the past few years [3], bed-nets were still desirable and their use was stated to be maintained.

Awareness of malaria and its symptoms was high among the caretakers interviewed. Susceptibility to malaria was perceived as low, especially in the dry seasons. Despite the perception that malaria could develop into a severe illness that could lead to disability and even death, the caretakers were confident that the risk of severity remained low as long as prompt appropriate treatment was sought. The acknowledgement of severe forms of malaria and the importance of treatment illustrate a better understanding of malaria than what was previously shown in coastal Tanzania [29]. In a review of the HBM from the 80s, Janz and Becker showed that perceived susceptibility was especially important in preventive health behavior, whereas perceived severity was the least powerful predictor and was more related to sick-role behavior and, therefore, less relevant for a preventive behavior [30]. Thus, the low perceived susceptibility, more than the low perceived severity might have a negative influence on bed-net usage.

Children, who were viewed as more susceptible to mosquito bites and malaria, were prioritized when it came to bed-net usage. Although prioritizing bed-net usage in small children is contrary to previous findings from a randomized control trial in Kenya where children were less likely to use bed-nets compared to older individuals [10], our finding does concur with more recent findings where under-five children were more likely to use bed-nets [12,13]. This is probably due to the delivery systems in recent years that have focused on distributing bed-nets to the vulnerable under-five children and pregnant women, which was also the case in the free distribution campaign in Zanzibar.

Benefits of bed-nets included malaria prevention as well as protection against mosquito nuisance, which has also been reported from Kenya [31,32]. In this study, due to the strong association made between mosquito bites and malaria, it was difficult to differentiate between the two benefits. Nonetheless, it was common that protection against mosquito bites was described independently of malaria transmission, and thus served as an additional benefit.

Although caretakers were well aware of the fact that mosquitoes transmitted malaria, other causes of malaria were also reported. Beliefs in alternative causes of malaria and their prevention could potentially lower the perceived benefit of bed-nets for malaria prevention [16,31,32]. In this study, the women in particular mentioned several alternative causes of malaria (i.e. dirt, poor hygiene, diet, etc.) and described prevention strategies against these causes. While the alternative causes mentioned cannot actually cause malaria, they may lead to other illnesses with similar symptoms, such as gastroenteritis. The fact that the female informants mentioned alternative causes more often than men could indicate that women are more exposed to both seeing different illnesses in children as well as to receiving health education messages provided by health workers, often resulting in a mix of biomedical and local pre-existing ideas and logics [19,33].

While complementary interventions for malaria and mosquito prevention such as IRS might lower the perceived benefit of bed-nets, the caretakers in this study did not see them as substitutes for the long-lasting physical barrier that a bed-net provides. This could partly be explained by the common belief that the insecticide used in both ITNs and IRS only affects the malaria-carrying mosquitoes and that other mosquitoes were perceived to be resistant; thus bed-nets were seen as a necessary barrier against all other mosquito types and insects. Additionally, the perceived short-lived effect of IRS hinders its eligibility to replace bed-nets; findings which concur with those found in a recent study in Mozambique, where IRS was perceived to have similar limitations [34].

As was previously documented by Winch et al. (1994) in Tanzania, seasonal usage of nets was attributed to the inconvenience of sleeping under the net in the heat, the perceived low mosquito density and perceived low risk of malaria in the dry seasons [11]. The discomfort of sleeping under the net during the hot season was also previously shown in Kenya [10]. While the heat barrier could be reduced by the use of nets with a large mesh size, a study that was conducted in the Solomon Islands demonstrated that large mesh-size was a cause of concern to the users who believed that mosquitoes might more easily penetrate the net [17]. While this concern, which has also been documented previously in Zanzibar [7], was raised in our study, the large mesh size was also acknowledged as a positive characteristic of LLINs as a way to increase ventilation and reduce heat. The finding that continuous use of bed-nets was more common in children suggests that the perceived higher risk of malaria in children outweighed the caretakers' perceived discomfort of being hot. According to Janz and Becker, the perceived barrier component was the most powerful single predictor among the HBM elements; and although benefits were important, they more strongly predicted sick-role behavior than preventive health behavior [30].

The high cost of the bed-nets was identified as a barrier for bed-net ownership. Although all informants received at least one free LLIN, the cost barrier would need to be addressed to ensure sustainable bed-net usage when bed-nets need to be replaced. Since this study took place just over a year after the LLIN distribution, and since the expected lifespan of the LLINs is about 4–5 years, cases in which the LLINs had been discarded or worn out were not encountered. This, however, can be expected to happen with time. The cost barrier is especially relevant since the low malaria transmission may reduce donors’ willingness to continue free distributions of bed-nets in Zanzibar in the future. Therefore, strategies to provide subsidized and affordable nets through the private and public sector should be implemented both during periodical free distributions as a "keep-up" strategy [35] and as a sustainable delivery mechanism when future funding for free distribution campaigns ebb out.

Women in this study described having high self-efficacy and decision-making power concerning bed-net use for children and they were recognized as a source of knowledge on health issues. The self-efficacy component was added to the HBM in the 80s, and is especially relevant when applied to long-term behavioral changes [36]. Therefore, it can be expected that women’s high self-efficacy and strong decision-making power are likely to facilitate regular bed-net use in small children.

Theoretical frameworks are useful in planning and designing health interventions, with the HBM being one of the most widely used theories to explain health service uptake and behavior [37]. Although the model has been used extensively, it has been criticized for simplifying health-related representational processes and solely focusing on individuals' attitudes and beliefs without taking into account social, economic and even emotional factors that may also influence health-related actions. In addition, there is dispute as to which of the HBM components have more influence on health-behavior and what the relationship between these components are [21,24]. In this study, the HBM was used as the framework to understand the community attitudes, perceptions and beliefs on malaria and bed-nets and not to describe the association between these beliefs and the actual behavior of caretakers.

Limitations of this study included the fact that the interviews were conducted by foreign researchers using interpreters. The presence of a foreigner may have compromised the comfort of the informant during the interview. Although mitigated by using an interviewer of the same gender as the informant, as well as adhering to appropriate dress-code, this limitation may have increased desirability bias, whereby the informants answered less truthfully in order to please the interviewer. Trustworthiness could also have been affected by the interpreter who may impact the findings [38]. This was mitigated by training the interpreter on the interview guide and by transcribing only the Kiswahili sections of the interview. In this way the text included only what the informant had been asked and what the informant had answered regardless of the original question asked by the researcher.

Also, due to logistical constraints, the first contact with the informants was handled by the Shehas (village leaders). Although the Shehas were asked to introduce the study as a discussion around children's health, the researchers did not have direct control over how the study was presented to the informants. An additional concern is that the interviewees were selected due to their extensive answers during a previous survey. Therefore, there may have been a selection bias where more empowered women were chosen. This could have overestimated the observed high self-efficacy and decision-making power, and this finding is probably not transferable to all Zanzibari women.

Despite the limitations, this study gives an overview of the perceptions of malaria and bed-net use in Zanzibar. It shows that, although malaria is no longer viewed as a major health issue, seasonal usage of bed-nets might be sustained due to the perceived benefit of bed-nets in reducing mosquito bites, especially during the rainy season. This study, however, did not link caretakers' perceptions to actual use, and therefore sustained bed-net usage despite low risk perceptions should be further investigated through quantitative surveys.

## Conclusions

Despite the reduction in malaria incidence and the resulting low malaria risk perceptions among caretakers, the benefit of bed-nets as the most proficient protection against mosquito bites upholds their use. This, in combination with the high perceived self-efficacy of caretakers, supports bed-net usage, while seasonality interrupts consistent use. High effective coverage of bed-nets could be further improved by reinforcing the benefits of bed-nets, addressing the seasonal heat barrier by using nets with larger mesh sizes and ensuring high bed-net ownership rates through sustainable and affordable delivery mechanisms.

## Competing interests

The authors declare that they have no competing interests.

## Authors' contributions

**NB** participated in the conception and design of study tools, coordinated the field work, carried out data analysis and interpretation of data, drafted the paper and revised the paper. **ASA** participated in the conception and design of the study and revised the paper. **HE** conceived of the study, designed the study tools, carried out the female interviews, participated in data analysis and interpretation of data, and revised the paper. **AJ** conceived of the study, designed the study tools, carried out the male interviews, participated in data analysis and interpretation of data, and revised the paper. **FMA** participated in the design of the study tools, carried out the female interviews, and revised the paper. **GR** participated in the design of the study tools, field work coordination, and revised the paper. **AKA** field work coordination, and revised the paper. **FW** revised the paper. **AB** participated in analysis and interpretation of data, participated in drafting and revising the paper. **KK** participated in the conception and design of the study, design of study tools, analysis and interpretation of data, participated in drafting and revising the paper. All authors read and approved the final manuscript.

## Authors' information

**NB** MPH, is a PhD student at the Division of Global Health (IHCAR), Department of Public Health Sciences, Karolinska Institutet, Stockholm, Sweden**. ASA** MPH, Manager of Zanzibar Malaria Control Programme (ZMCP), Ministry of Health, Zanzibar, Tanzania**. HE** Master of Science in Psychology, is a licensed clinical psychologist at Cereb AB, Stockholm, Sweden. **AJ** Master of Science in Psychology, is a licensed clinical psychologist at OCD-Centret AB, Uppsala, Sweden. **FMA** Diploma in Community Health, Health Promotion Unit, Ministry of Health, Zanzibar. **AKA** BSc. Med. Lab, Malaria Control Programme (ZMCP). Ministry of Health, Zanzibar, Tanzania. **FW** MD, Prof, Department of Epidemiology & Biostatistics, Makerere University School of Public Health, Kampala, Uganda. **GR** MD, MPH, 4^th^ year student of specialization program in General Practice, EAP Canet Unitat Docent, Girona, Spain and Part-time researcher, Healthcare Research Support Unit Metropolitana Nord. IDIAP, Barcelona, Spain. **AB** MD, Prof, Malaria Research Unit, Department of Medicine Solna, Karolinska Institutet, Stockholm, Sweden**. KK** MSc, PhD, is a part-time researcher at the Division of Global Health (IHCAR), Karolinska Institutet, Stockholm, Sweden**;** honorary lecturer at Makerere University School of Public Health, Kampala, Uganda, and full time Regional Coordinator at Malaria Consortium, Kampala, Uganda.

## Pre-publication history

The pre-publication history for this paper can be accessed here:

http://www.biomedcentral.com/1471-2458/12/606/prepub
